# Effect of Surfactants on Posaconazole Crystallization and Polymorphism: Tween 80 vs Span 80

**DOI:** 10.1021/acs.cgd.5c01016

**Published:** 2025-08-12

**Authors:** Kennedy A. Borchardt-Setter, Xin Yao, Rose K. Cersonsky, Torsten Stelzer, Shuang Chen, Ahmad Sheikh, Geoff G. Z. Zhang, Lian Yu

**Affiliations:** † School of Pharmacy, 5228 University of Wisconsin–Madison, Madison, Wisconsin 53705, United States; ‡ Department of Chemical and Biological Engineering, University of Wisconsin–Madison, Madison, Wisconsin 53706, United States; § Department of Pharmaceutical Sciences, University of Puerto Rico, Medical Sciences Campus, San Juan, Puerto Rico 00936, United States; ∥ Crystallization Design Institute, Molecular Sciences Research Center, University of Puerto Rico, San Juan, Puerto Rico 00926, United States; ⊥ Molecular Profiling and Drug Delivery, AbbVie, Inc., North Chicago, Illinois 60064, United States; # ProPhysPharm LLC, 250 Parkway Drive, Suite 150, Lincolnshire, Illinois 60069, United States; ¶ Department of Industrial and Molecular Pharmaceutics, Purdue University, West Lafayette, Indiana 47907, United States; ∇ Department of Chemistry, University of Wisconsin–Madison, Madison, Wisconsin 53705, United States

## Abstract

A surfactant is often present in a pharmaceutical formulation and can accelerate the crystallization of the active component. We investigate the effects of two common surfactants with different hydrophilic–lipophilic balance, Span 80 and Tween 80, on the crystallization of posaconazole (POS) in its two polymorphic forms (I and II). At 0–10 wt %, the two surfactants similarly increase the growth rates of both polymorphs, but have different effects on their nucleation rates, without exhibiting the proportional-modification behavior observed for many other amorphous systems. The classical nucleation theory (CNT) can quantitatively describe the nucleation kinetics in the pure POS melt and in the surfactant-doped melts, where the surfactants are treated as ideal diluents of POS. The two surfactants’ effects on the nucleation rate form a single trend in the CNT analysis, yielding the nucleus/liquid interfacial tension σ = 0.019 J/m^2^ for Form I and 0.012 J/m^2^ for Form II. The σ value of 0.019 J/m^2^ for Form I nucleating from a doped melt exceeds that for nucleating from a pure melt (0.012 J/m^2^), possibly reflecting a greater structural difference between a chemically pure crystal and an impure melt. This work has provided a strong test of the CNT by applying it to both pure and doped melts where the crystallization driving force is varied through temperature in the pure system and through concentration in the doped system, and yielded a simple picture for two chemically distinct surfactants on the crystallization in a polymorphic system.

## Graphical abstract

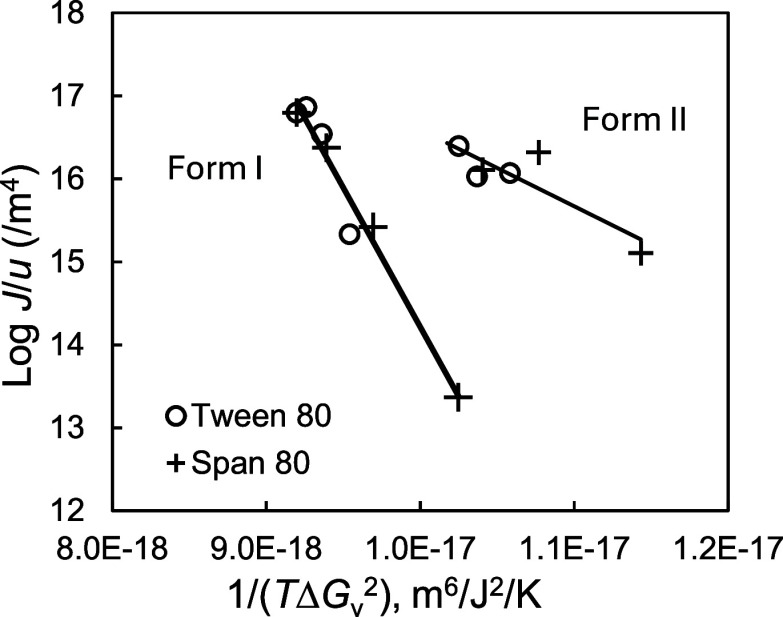

## Introduction

Amorphous solid dispersions (ASDs) are increasingly used to deliver poorly soluble drugs, taking advantage of an amorphous drug’s higher solubility than its crystalline counterpart^1,2^ and perhaps the efficient release of the active ingredient as nanoparticles.^3–6^ In addition to a drug and a polymer,^7–10^ an ASD often contains a surfactant for improving wetting and dissolution^11^ and for reducing the process temperature for a melt-extruded product.^12^ There is a strong current interest in the effect of a pharmaceutical surfactant on the crystallization of the active ingredient, since these excipients are usually liquids under ambient conditions and can increase the mobility of an ASD, causing faster crystallization.^13–17^ This is to be avoided since crystallization would eliminate the advantages of an ASD.

The crystallization process has two elemental steps, nucleation and growth, with each step having its unique kinetics and polymorphic dependence.^18–20^ In the presence of a second, noncrystallizing component, it is necessary to assess its effect on each elemental step to predict its overall effect. In this context, a useful recent finding is the proportional changes of the nucleation rate and the growth rate of an amorphous drug by a dopant at a relatively low concentration.^15,21,22^ Yao et al. report that near the glass transition temperature *T*_g_, a dilute surfactant increases the rates of nucleation and growth of the host by a similar factor, thus enabling the prediction of harder-to-measure nucleation rates from easier-to-measure growth rates.^15^ Similar simplification has been observed for polymers as dopants, where the polymer lowers the mobility of the host molecules and slows their nucleation and growth proportionally.^21–23^ In the framework of the Classical Nucleation Theory (CNT), which treats the nucleation rate as a product of a kinetic (mobility) factor and a thermodynamic factor, this simplicity arises if the dopant mainly modifies the kinetic factor but has little effect on the thermodynamic factor. It is of interest to learn how common this simplicity is and under what conditions it breaks down.

Many substances can crystallize as multiple polymorphs, and predicting which one does under a given condition has fundamental and practical importance.^24,25^ The polymorphism of a drug^26^ influences its solubility, bioavailability, manufacturability, and stability. At present, polymorphic selection in crystallization remains poorly understood. For example, knowing the crystal structures of two polymorphs allows prediction of many of their physical properties, but does not yet enable a confident prediction of which polymorph crystallizes when both are thermodynamically driven to do so. For crystallization from a pure melt, Gui et al. observed that the faster-nucleating polymorph tends to have higher enthalpy and lower density, but this is an empirical result and currently lacks a fundamental justification.^20^ They also noted that a fast-growing polymorph need not be fast-nucleating,^20^ again highlighting the importance to consider both nucleation and growth in predicting the outcome of crystallization. This already complex problem becomes even more challenging in the presence of a second component. Progress in this area calls for experimental investigations of crystallization in polymorphic systems and interpretation of the results with the aid of crystal structures and computer simulations.

This work investigates the effect of two structurally related surfactants on the melt crystallization and polymorphic selection of posaconazole (POS). A poorly soluble antifungal (see Scheme 1 for its structure), POS has served as a model for the studies of amorphous drugs.^27–30^ POS has two polymorphs, Forms I and II,^31^ and many solvated crystal forms.^32^ In the two polymorphs, the rod-like POS molecules adopt different conformations, with the difluorophenyl ring being bent relative to the long axis in Form I and extended in Form II. Both polymorphs are organized as tilted molecular layers, with the layers in Form I comprising molecules in antiparallel arrangement and those in Form II in parallel arrangement.^31^ The two polymorphs are enantiotropic, with Form I being more stable below 393 K and Form II being more stable above.^31^ Yao et al. have shown that the free surface of a POS melt nucleates a different polymorph (II) from its interior (I), a consequence of the preferred orientation of the molecules at the interface.^31^

**Scheme 1:**
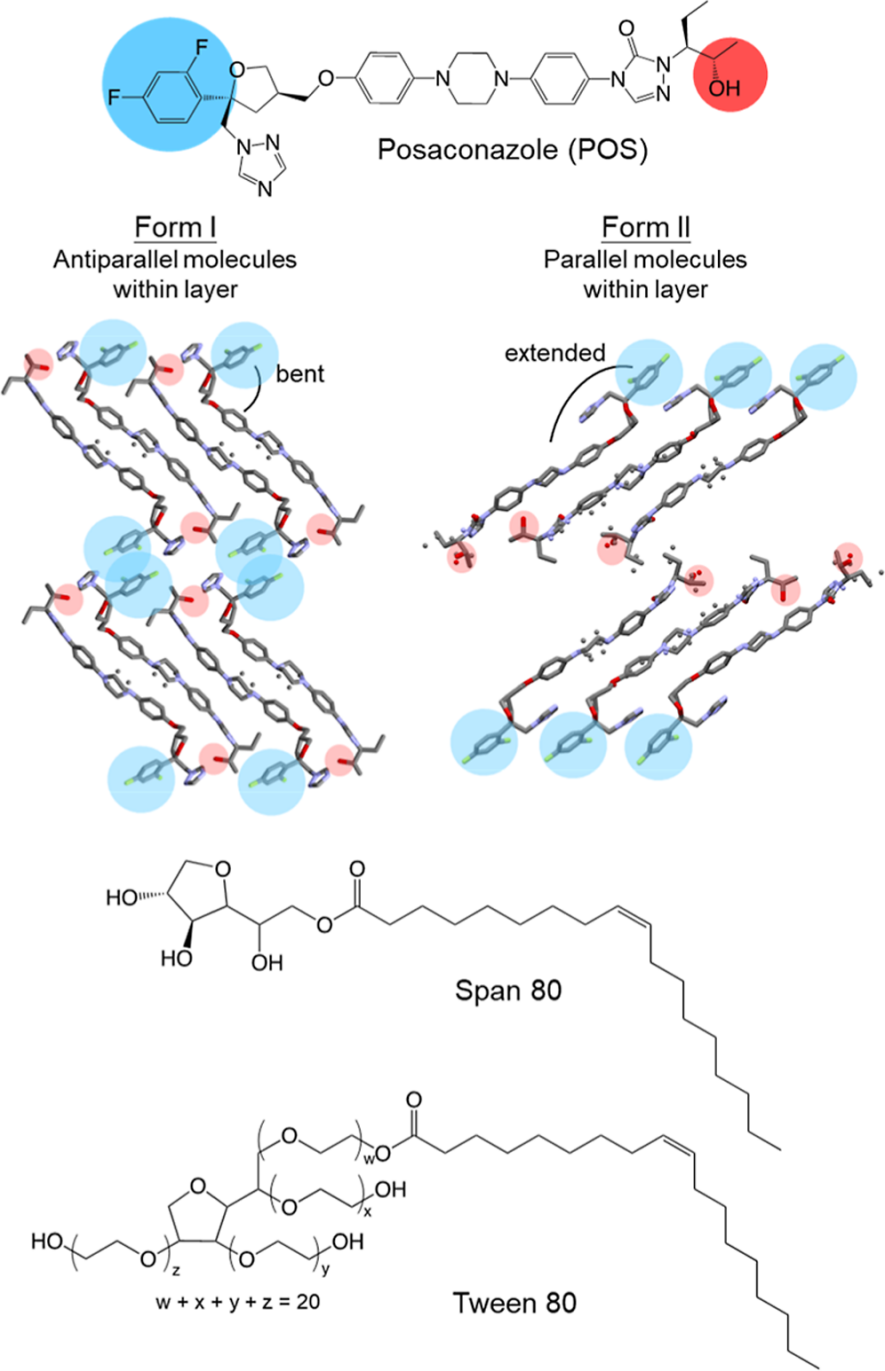
Molecular Structures of Posaconazole (POS), Span 80, and Tween 80^a^

a Under the molecular drawing of POS are the crystal structures of its two polymorphs (I and II).^28^ The “floating” atoms indicate positional disorder.

The two surfactants of this study, Tween 80 and Span 80 (Scheme 1), share the same hydrophobic tail but have different hydrophilic heads. With its larger hydrophilic head, Tween 80 has a higher Hydrophilic–Lipophilic Balance (HLB) value and is the more water-soluble. See Table 1 for some of the physical properties of the two surfactants. Tween 80 has a higher critical micelle concentration (CMC) in water but lower CMC in benzene. Despite its higher molecular weight, Tween 80 has lower viscosity and *T*_g_,^15^ a result of the high mobility of the PEO segments.

**Table 1:** Physical Properties of Tween 80 and Span 80^a^

	Tween 80 (polysorbate 80)	Span 80 (sorbitan monooleate)
molecular weight (g/mol)	1310	428.6
HLB	15.0	4.3
CMC in water (mM)	0.052	0.026
CMC in benzene (mM)^34^	4.0	9.5
viscosity at 25 °C (mPa s)	425	970 – 1080
*T*_g_ (K)^15^	201	212

a From ref 33 unless otherwise noted.

In this work, we find that Tween 80 and Span 80 (2–10 wt %) have similar enhancement effects on the growth rates of the two polymorphs of POS, but different effects on their nucleation rates, without exhibiting the proportional-enhancement behavior noted previously for many amorphous systems.^15^ The CNT can quantitatively describe the nucleation kinetics in the pure POS melt and in the surfactant-doped melts, where the surfactants are treated as ideal diluents of POS. Thus, despite their structural difference, Tween 80 and Span 80 have similar effects on the nucleation rate of POS when compared at the same mole fraction. This allows the nucleation rates in the two doped systems to be fitted together, yielding polymorph-specific nucleus/liquid interfacial tensions. While Form I is favored to nucleate from a pure melt, Form II nucleation becomes more favored with increasing dopant concentration. In the CNT framework, this arises from a lower nucleus/liquid interfacial tension for Form II. This work provides a strong test of the CNT by simultaneously applying it to pure and doped melts and uncovers a surprising simplicity for two chemically distinct surfactants as dopants.

## Materials and Methods

### Materials

Posaconazole (POS) was a gift from Merck. Span 80 and Tween 80 were purchased from Sigma-Aldrich. All materials were used as received.

### Drug-Surfactant Mixtures

The mixtures containing 10 and 20 wt % surfactant in POS were prepared by grinding weighed ingredients in a mortar with a pestle with trace ethanol, which was evaporated overnight at ambient conditions. The 2 and 5 wt % mixtures were prepared by diluting a 10 wt % mixture with additional POS using the same solvent-grinding procedure.

### Differential Scanning Calorimetry

A TA Q2000 differential scanning calorimeter (DSC) was used to measure the glass transition temperatures and the melting points. About 3–4 mg of a sample was loaded in a crimped aluminum pan and analyzed under 50 mL/min N_2_ purge. In a typical temperature program, the sample was heated at 10 K/min to 458 K, cooled at 5 K/min to 193 K, and reheated at 10 K/min to 458 K. POS has been reported to be thermally stable up to 600 K.^35^ The *T*_g_ was determined as the onset of the glass transition during the second heating.

### Raman Microscopy

Raman spectra of microscopic crystals were measured with a LabRAM HR Evolution Confocal Raman Microscope with a 532 nm laser. The spectra were measured between 400 and 1400 cm^–1^ with an acquisition time of 10 s and 3 accumulations per scan. A grating of 1800 gr/mm was employed, and the laser power was 3.2%. Crystallized samples were analyzed after removing the top coverslip from a sandwich sample. The microscope objective used was 100× and provided a spatial resolution of approximately 1 μm.

### Powder X-ray Diffraction (PXRD)

PXRD patterns for the samples were collected using a Bruker D8 ADVANCE unit with a Cu Kα source operating at a tube load of 40 kV and 40 mA. Scans were taken between 5° and 40° (2θ) at 0.02° steps and a maximum rate of 1 s/step.

### Crystal Nucleation and Growth Rates

To measure the nucleation rate, a two-stage bulk nucleation process was used.^20^ Briefly, a liquid film between two coverslips was prepared by melting the powder on one coverslip and covering the melt with another. The thickness of the resulting sandwiched film was uniform and determined by its mass, lateral area, and density. The sample was allowed to nucleate at 353 K controlled by a hot stage (Linkam THMS) for a chosen time *t*_n_ and heated to 403 K for 1 min to grow the nucleated crystal to visible size. The sample was quenched to room temperature and the number of crystals was counted through a light microscope (Olympus BX53). Each experiment was repeated in triplicate to yield the nucleation rates reported. To measure the crystal growth rate, the sample was placed on the hot stage at a set temperature and the advancing speed of a crystal front was measured through the light microscope.

## Results

### Crystal Nucleation and Growth in Pure POS Melt

To orient this study of the doped systems, we briefly review the nucleation and growth rates in a pure POS melt from previous work in this lab^31^ and provide additional analysis needed for later discussion. Figure 1a shows the crystal nucleation rate *J* in a pure POS melt as a function of temperature. At present, only Form I’s nucleation rate has been measured in a pure melt and Form II’s rate is too slow to be measured. Figure 1b shows the crystal growth rates *u* of the two polymorphs in a POS melt. Despite its slow nucleation, Form II’s growth rate was measurable above 370 K by initiating its growth with seeds obtained by microdroplet melt crystallization.^31^ Below 370 K, it is difficult to measure Form II’s growth rate because Form I crystals nucleate on Form II crystals (cross nucleation between polymorphs^36^), blocking further growth. Where comparison is possible (>370 K), Form II grows slower than Form I, by roughly a factor of 2.

**Figure 1:**
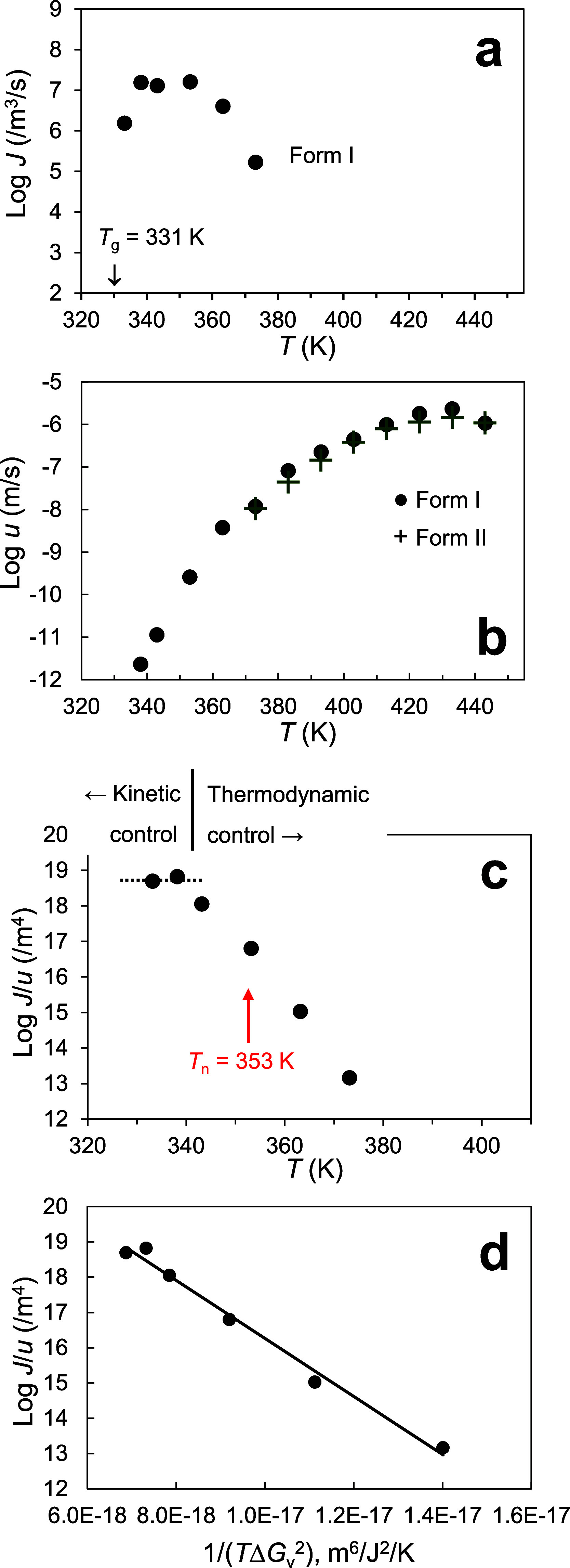
Rates of (a) crystal nucleation and (b) growth in pure POS melt from ref 31. (c) *J*/*u* vs *T* plot indicating the kinetic and thermodynamic regimes and the nucleation temperature of this work (353 K). (d) Log *J*/*u* vs 1/(*T*Δ*G*_v_^2^) plot. Linearity of this plot indicates that the CNT accurately describes the nucleation process, with σ = 0.012 J/m^2^ for the nucleus/liquid interfacial tension.

Figure 1a,b show that the nucleation rate of Form I peaks at a lower temperature (350 K) than its growth rate (430 K). This is a familiar result,^18–20^ reflecting the greater supercooling needed to create a crystal embryo than to grow an existing crystal. Below 350 K, *J* and *u* both fall with cooling and do so at comparable rates. To test this behavior further, in Figure 1c, the ratio *J*/*u* is plotted against *T*, and we observe that with cooling, *J*/*u* initially increases and then plateaus. The plateau indicates a proportional decrease of *J* and *u*, and defines the low-temperature region where both nucleation and growth are kinetically controlled with a similar kinetic barrier. This behavior has been observed for other systems,^18,20^ and indicates that *u* can be used to represent the kinetic factor in the CNT as discussed below.

According to the CNT,^37,38^ the nucleation rate is given by

1 $$J=k_{J}\;\exp\left(-W_{c}/kT\right)$$

where *k*_J_ is the kinetic factor specifying the attempt frequency at which molecules join the nucleus, and *W*_c_ represents the thermodynamic barrier for creating a critical nucleus. For a spherical nucleus, $W_{c}=\frac{16\pi }{3}\frac{\sigma^{3}}{\Delta G_{v}^{2}}$ , where σ is the nucleus/liquid interfacial tension and Δ*G*_v_ = *G*_L_ – *G*_C_ is the driving force of crystallization. The kinetic factor *k*_J_ is usually taken to be proportional to some measure of the nucleating liquid’s mobility (e.g., diffusivity). Huang et al. have proposed that the crystal growth rate *u* be used for this purpose because it better represents the dynamics at the nucleus/liquid interface than a bulk property like viscosity and is often measured along with *J* in the same study.^19^

Following Huang et al., we test the validity of the CNT in describing nucleation in a POS melt by plotting ln (*J*/*u*) against 1/(*T*Δ*G*_v_^2^). Linearity of this plot would support the theory’s validity and the resulting slope would yield the σ value. Figure 1d shows that the plot is indeed linear (*R* ^2^ = 0.99), from which we obtain σ = 0.012 J/m^2^. For this plot, Δ*G*_v_ is calculated from Form I’s temperature and enthalpy of melting, *T*_m_ and Δ*H*_m_, assuming the liquid-crystal heat capacity difference is given by (0.003/K) Δ*H*_m_.^39^ The CNT analysis described above has been performed by Song et al.^23^ using the same data (Figure 1a,b), but a different expression for Δ*G*_v_; it yielded the same σ value within two decimal places. The σ value is comparable to those of other molecular liquids^18,20,22^ and will be compared to the values for doped POS melts below.

In this work, nucleation in doped POS melts was studied at 353 K, which is near and slightly above the peak temperature for nucleation of Form I (Figure 1a). This temperature is in the regime of thermodynamic control (arrow in Figure 1c), meaning that if the dopant significantly alters the driving force of crystallization (equivalent to changing the temperature of a pure melt), the nucleation rate should change by a different factor from the growth rate. But if the dopant only modifies the mobility but not the crystallization driving force, the nucleation rate and the growth rate are expected to change by the same factor, showing the “equal enhancement and suppression” behavior.^15,21,22^

### Component Miscibility and Phase Relations

As preparation for studying the dopant effect, we establish in this section whether the dopants are dissolved in the host or phase separated, as well as how the two polymorphs are related to each other and with the amorphous phase in terms of thermodynamic stability. Figure 2 shows the DSC traces in the glass transition region for POS containing Tween 80 and Span 80. Since the surfactant has a significantly lower *T*_g_ than POS (*T*_g_ = 201 K for Tween 80,^15^ 212 K for Span 80,^15^ and 333 K for POS), their dissolution in POS should lower its *T*_g_. In Figure 2a,b, the step-like event is the glass transition, and we define its onset as *T*_g_. Figure 2c plots the *T*_g_ of each binary system as a function of the surfactant concentration. For each system, *T*_g_ decreases with the increase of surfactant concentration and the decrease is faster for Tween 80 as dopant. The latter result is consistent with the lower *T*_g_ of Tween 80. Overall, the DSC results indicate that both surfactants are miscible with POS at the concentrations used for our nucleation studies (2–10 wt %).

**Figure 2:**
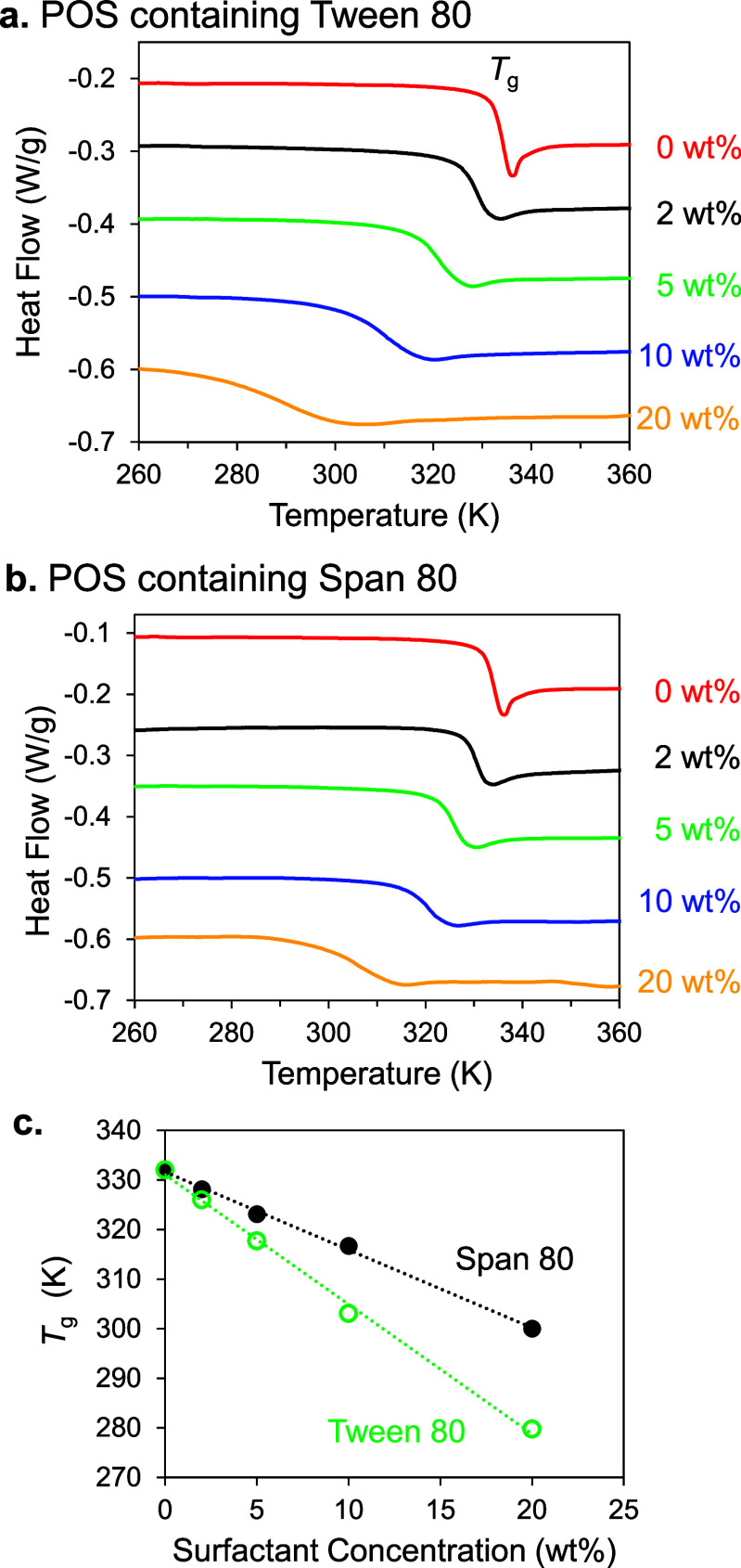
DSC traces of POS containing (a) Tween 80 and (b) Span 80 at the concentrations indicated. (c) *T*_g_ plotted against surfactant concentration.

Figure 3 shows the DSC traces for the systems near the crystal-melting region. For pure POS (Figure 3a), the full traces show the melting endotherms of the two polymorphs (I and II), with II melting at a higher temperature. Form I shows a reversible solid-state transition (I ↔ I′) prior to melting. Figure 3b shows that doping POS with a surfactant depresses the crystal melting points, as expected. The melting-point depression further supports the miscibility of the components. For each polymorph, 10 wt % Span 80 causes larger melting-point depression than 10 wt % Tween 80. This is expected from the colligative nature of the melting-point depression phenomenon: Span 80 has a lower molecular weight than Tween 80 (428.6 vs 1310 g/mol), and at the same wt %, has a higher molar concentration and should cause larger melting-point depression.

**Figure 3:**
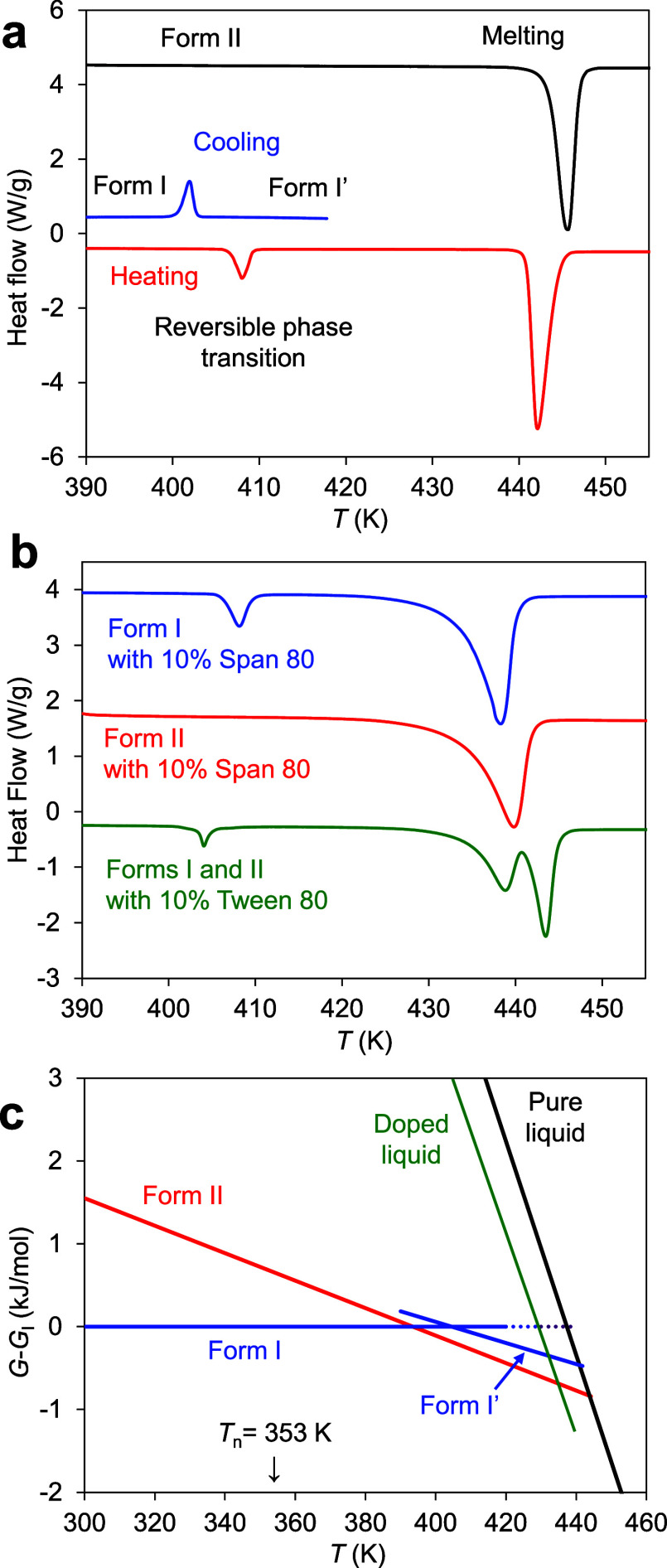
DSC traces of POS polymorphs near their melting regions. (a) Pure POS. (b) POS containing 10 wt % Tween 80 or Span 80. (c) Phase diagram indicating the relative stability of POS polymorphs and their melt. A dopant decreases the drug’s chemical potential in the melt, as illustrated. The down arrow marks the nucleation temperature of this work (353 K), at which Form I is more stable than Form II.

Figure 3c shows the relative Gibbs free energies (chemical potentials) of POS in its polymorphs and in the melt, either pure or doped. Forms I and II are related enantiotropically, with Form I being more stable below 393 K. Above this temperature, Form I exhibits a solid-state transition I ↔ I′. The intersection of the crystal lines with the liquid line defines the crystal melting points. In the presence of a dissolved dopant, the drug’s chemical potential in the liquid phase decreases, as shown, but unless the dopant dissolves in the crystals to form a solid solution, its chemical potential remains constant in the solid phase. At the nucleation temperature of this study (353 K), Form I is the stable polymorph and Form II is the metastable polymorph.

### Effect of Tween 80 on Crystallization and Polymorphism of POS

We now report the effect of each surfactant (Tween 80 or Span 80) on the crystallization and polymorphism of POS. We first consider each dopant separately and then compare them. Yao et al. reported that crystallization from a pure POS melt preferentially yields one polymorph, Form I.^31^ In this work, however, we observed that two polymorphs of POS crystallize from a surfactant-doped melt. Figure 4a shows a photomicrograph of a nearly fully crystallized melt of POS that contained 10 wt % Tween 80. The crystallization temperature was 353 K. The crystals grew as smooth spherulites, but at two distinct growth rates, and as a result, the slower growing spherulites were often engulfed by the faster growing ones; see Figure 4a for examples. By Raman microscopy, the two types of spherulites were shown to be the two polymorphs of POS (Figure 5), with the faster-growing crystals being Form I and the slower-growing being Form II. For this analysis, the authentic sample of each polymorph was certified by PXRD and measured to obtain the reference spectrum. The as-purchased POS powder was the authentic Form I; the authentic Form II was prepared by crystallizing a pure melt with an exposed free surface.^31^

**Figure 4:**
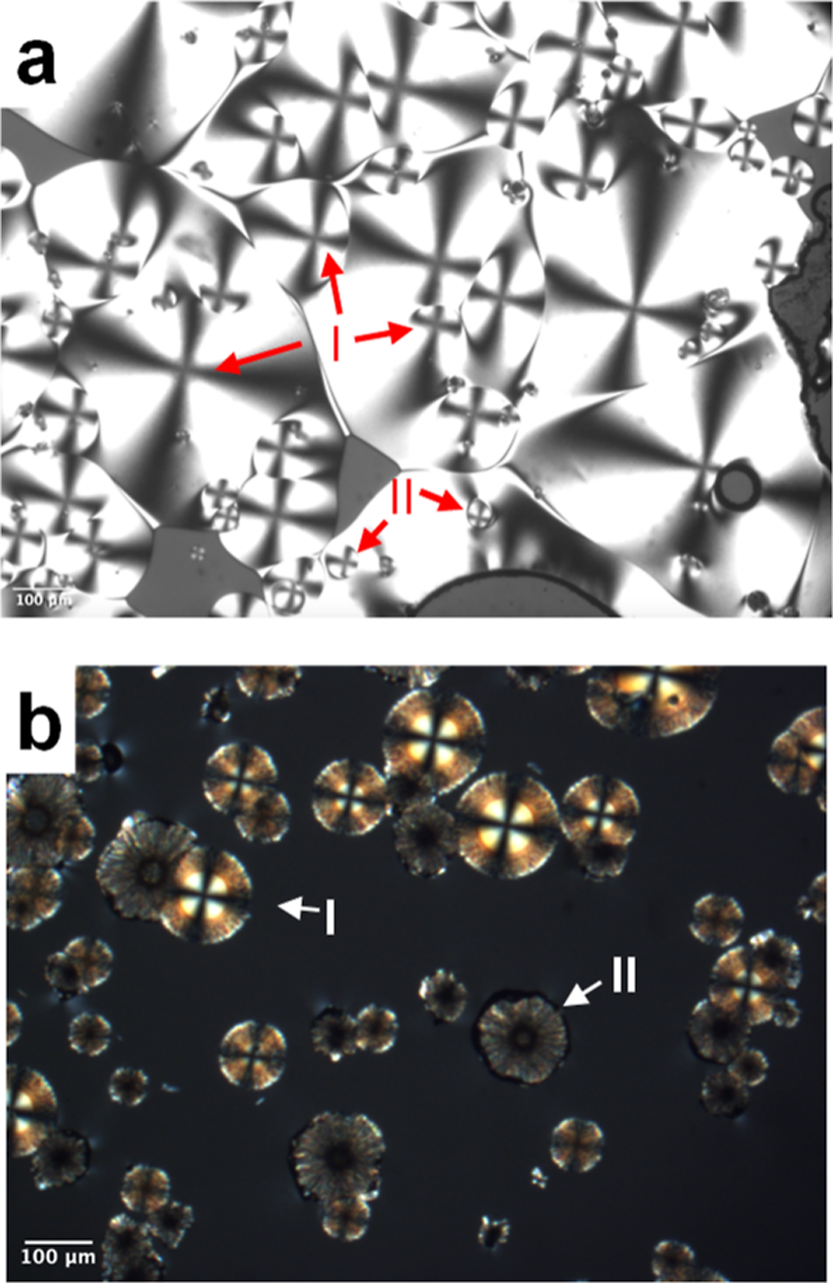
(a) Crystallization of two polymorphs from a POS melt containing 10 wt % Tween 80 at 353 K. The polymorphs have different growth rates, with Form I growing faster than and often engulfing Form II. (b) Appearance of the two polymorphs in a two-stage experiment for measuring their nucleation rates. This sample was nucleated at 353 K for 100 min and grown at 403 K for 1 min. The polymorphs were confirmed by their Raman spectra (Figure 5).

**Figure 5:**
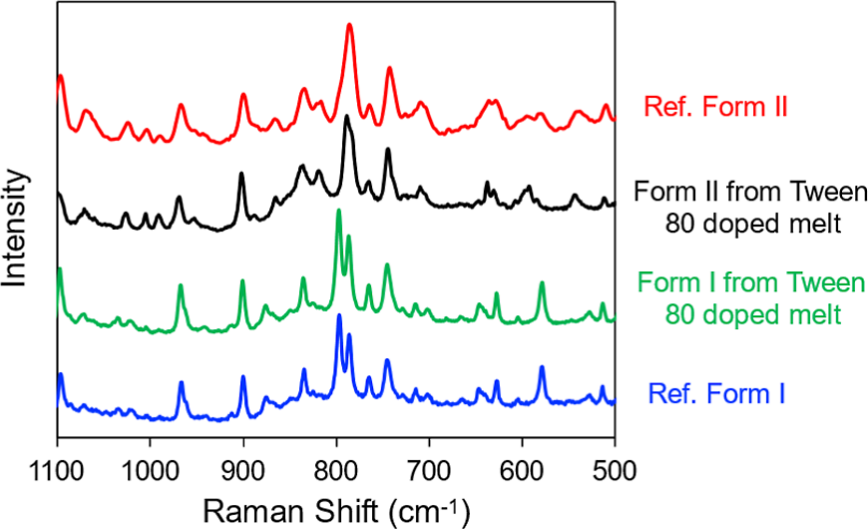
Identifying POS polymorphs I and II from Tween 80 doped melt by Raman microscopy.

Figure 4b shows the appearances of the two polymorphs in a two-stage experiment for measuring their nucleation rates. This sample was nucleated at 353 K and grown at 403 K. The observed crystals showed two morphologies corresponding to the two polymorphs. Form I crystals featured smooth edges and Form II crystals showed rougher edges. Some Form I crystals contained a central region grown at 353 K prior to heating at 403 K with a Maltese cross when viewed between crossed polarizers; others lacked this central region because the growth occurred mostly at 353 K. Again, Raman spectra (Figure 5) were used to identify these polymorphs. Although the two polymorphs have very different growth rates at 353 K, their growth rates are similar at 403 K, reflecting the changes of the thermodynamic and kinetic factors controlling crystal growth. As a result, no engulfment phenomenon was observed.

Figure 6 illustrates the measurement of crystal nucleation rates in a POS melt containing Tween 80 utilizing a two-stage method. In this method, nuclei are formed at a lower temperature (353 K in this case) for a set time *t*_n_ and grown to visible size at a higher temperature (403 K in this case) for counting. Figure 6a illustrates the increase of crystal density after increasing *t*_n_ from 10 to 100 min. Figure 6b plots the volumetric density of counted crystals against *t*_n_ for both polymorphs. Each plot shows an initial period of slower nucleation and a subsequent period of faster nucleation where the nuclei density increases linearly over time. The initial period is the induction time for nucleation and the subsequent period is the steady state of nucleation. The slope of the plot at the steady state is the reported nucleation rate.

**Figure 6:**
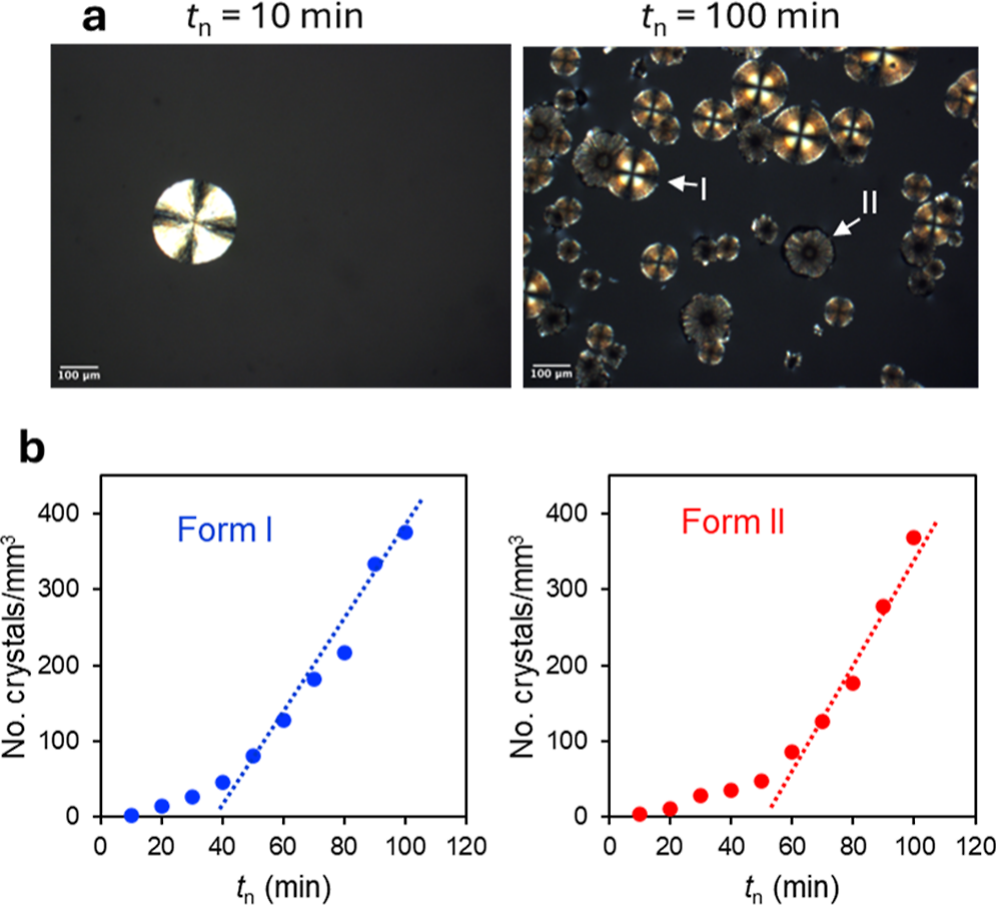
Measuring nucleation rates in POS doped with 10 wt % Tween 80 using a two-stage method. (a) Typical images showing higher crystal density after increasing the nucleation time at 353 K from 10 to 100 min followed by the same growth time (1 min) at 403 K. (b) Crystal density plotted against *t*_n_. The trendlines indicate the slope at the steady state, which is the nucleation rate.

Figure 7a shows the nucleation rates of the two polymorphs of POS (I and II) from a melt containing Tween 80. The rate of Form I nucleation from a pure POS melt is from ref 15; the Form II nucleation rate could not be measured and is presumably slower. With increasing Tween 80 concentration, the Form I nucleation rate increases and plateaus, while the Form II nucleation rate shows a steady increase, catching up with the Form I rate at 10 wt %.

**Figure 7:**
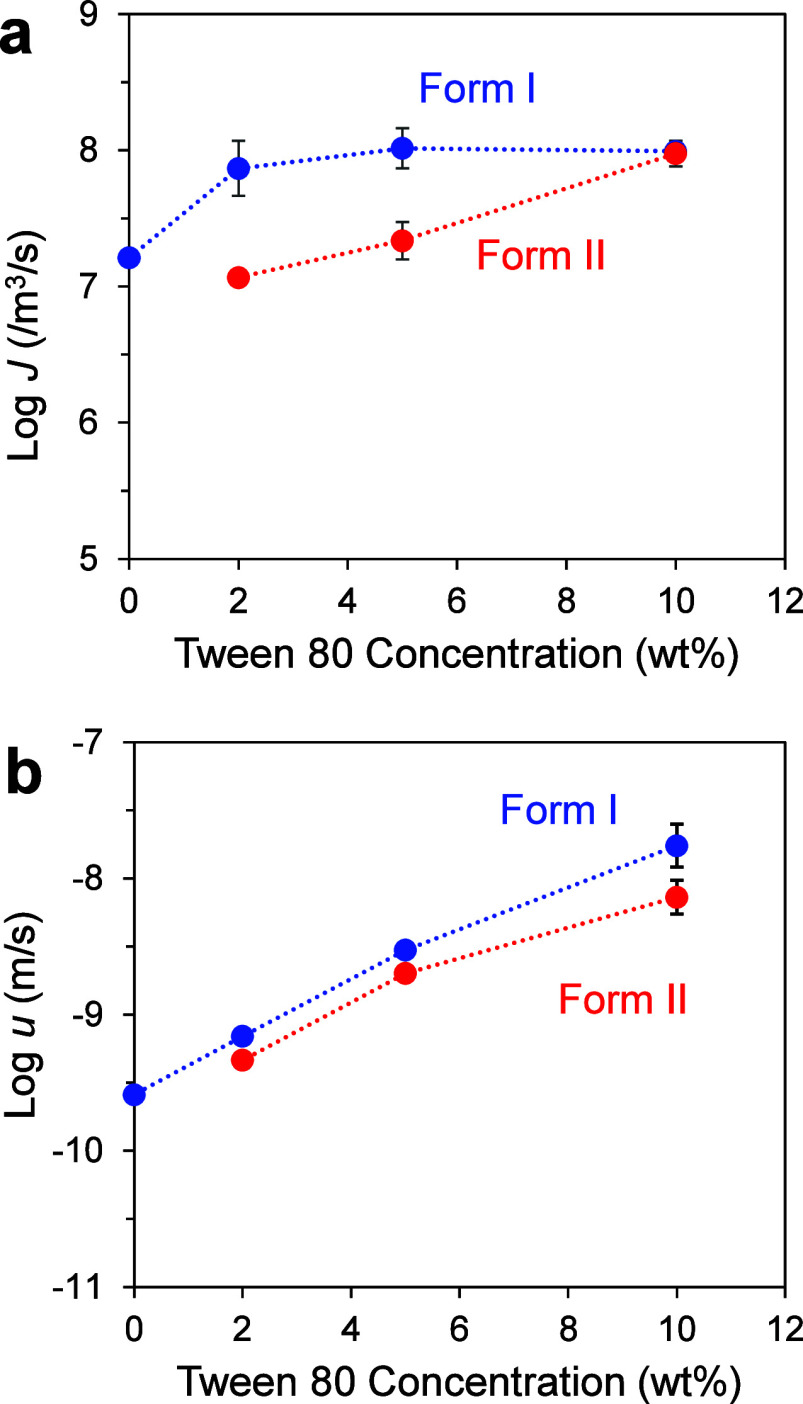
(a) Nucleation rates and (b) growth rates of POS polymorphs at 353 K as functions of Tween 80 concentration.

Figure 7b shows the growth rates of the two polymorphs in a POS melt containing Tween 80. The measurement was performed at 353 K, the same temperature at which nucleation rates were measured (Figure 7a). Both polymorphs grow faster as Tween 80 concentration increases. For Form I, the effect was 2 orders of magnitude in the concentration range investigated. Together, the data in Figure 7 do not show the simplicity of proportional changes of the nucleation and growth rates reported for other systems.^21,23^ This difference will be discussed later.

Figure 8 presents the result of a “thickness test” to determine whether the nucleation observed is a volume process, as expected for homogeneous nucleation, or a surface-induced process. In this test, the number of crystals that formed in a liquid film confined under a 12 mm diameter coverslip was recorded. Each film was nucleated at 353 K for 70 min and grown at 403 K for 1 min, and the film thickness was varied between samples. For a volume process, the number of crystals should be proportional to the liquid-film thickness; for a surface-induced process, the number should remain constant. Figure 8 shows that the number of crystals observed is proportional to the liquid-film thickness, and the trend holds for both Form I and Form II. These results indicate that the nucleation process observed is a volume process and justifies its analysis later as a homogeneous nucleation process.

**Figure 8:**
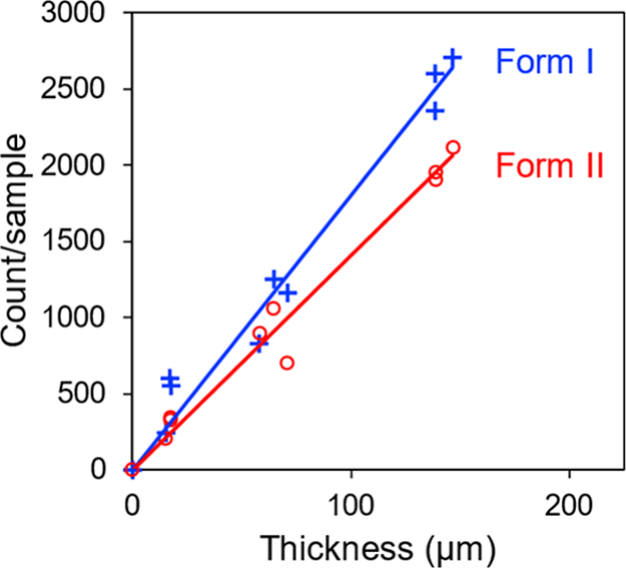
Thickness test to demonstrate that crystal nucleation observed in POS doped with 10 wt % Tween 80 is a volume process.

### Effect of Span 80 on Crystallization and Polymorphism of POS

The same protocol described above for POS containing Tween 80 was used to characterize the effect of Span 80 on the crystallization of a POS melt. In this case, we also observed two polymorphs crystallizing from the doped melt. Figure 9a shows the different morphologies of the two polymorphs that formed at 353 K. Form II crystals have a spiked morphology; Form I crystals have smoother edges. Again, Raman spectroscopy was used to identify these polymorphs (Figure 10). Figure 9b shows the appearances of the two polymorphs in a two-stage experiment for measuring their nucleation rates. This sample was nucleated at 353 K and grown at 403 K. Form II crystals were spherulites with a Maltese Cross when viewed between crossed polarizers. Form I crystals had more irregular shapes and rougher edges.

**Figure 9:**
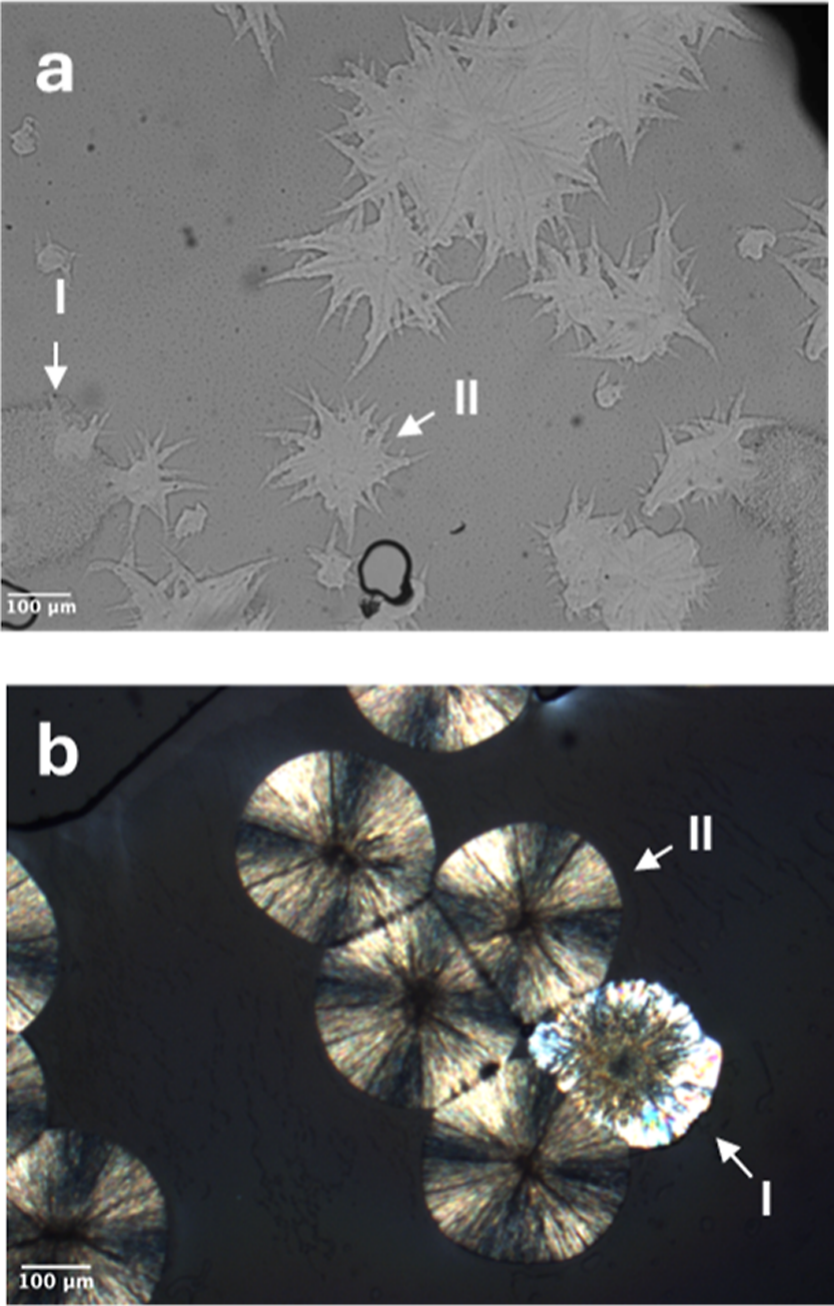
(a) Crystallization of two polymorphs from a POS melt containing 10 wt % Span 80 at 353 K. The polymorphs have different morphologies. (b) Appearance of the two polymorphs in a two-stage experiment for measuring nucleation rates. This sample was nucleated at 353 K for 100 min and grown at 403 K for 1 min. The polymorphs were confirmed by their Raman spectra (Figure 10).

**Figure 10:**
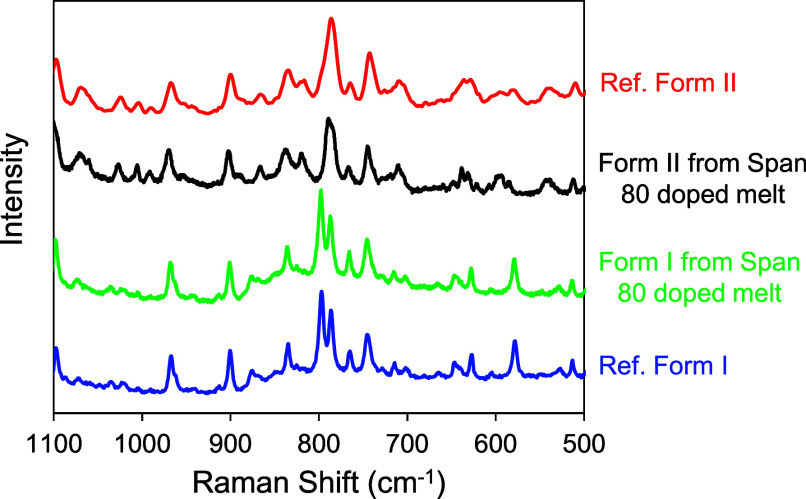
Identifying POS polymorphs I and II from Span 80 doped melt by Raman microscopy.

Figure 11 illustrates the measurement of crystal nucleation rates in POS doped with Span 80 again utilizing the two-stage method. The sample was nucleated at 353 K for different nucleation times *t*_n_ as indicated and then grown at 403 K for 1 min. Figure 11a illustrates the increase of crystal density as a result of increasing *t*_n_. Figure 11b plots the volumetric density of counted crystals against *t*_n_. As in the case of Figure 6b, an induction period is observed, followed by the period of faster nucleation in which the crystal density increases linearly with time (steady state). The slope of the plot at the steady state is the nucleation rate.

**Figure 11:**
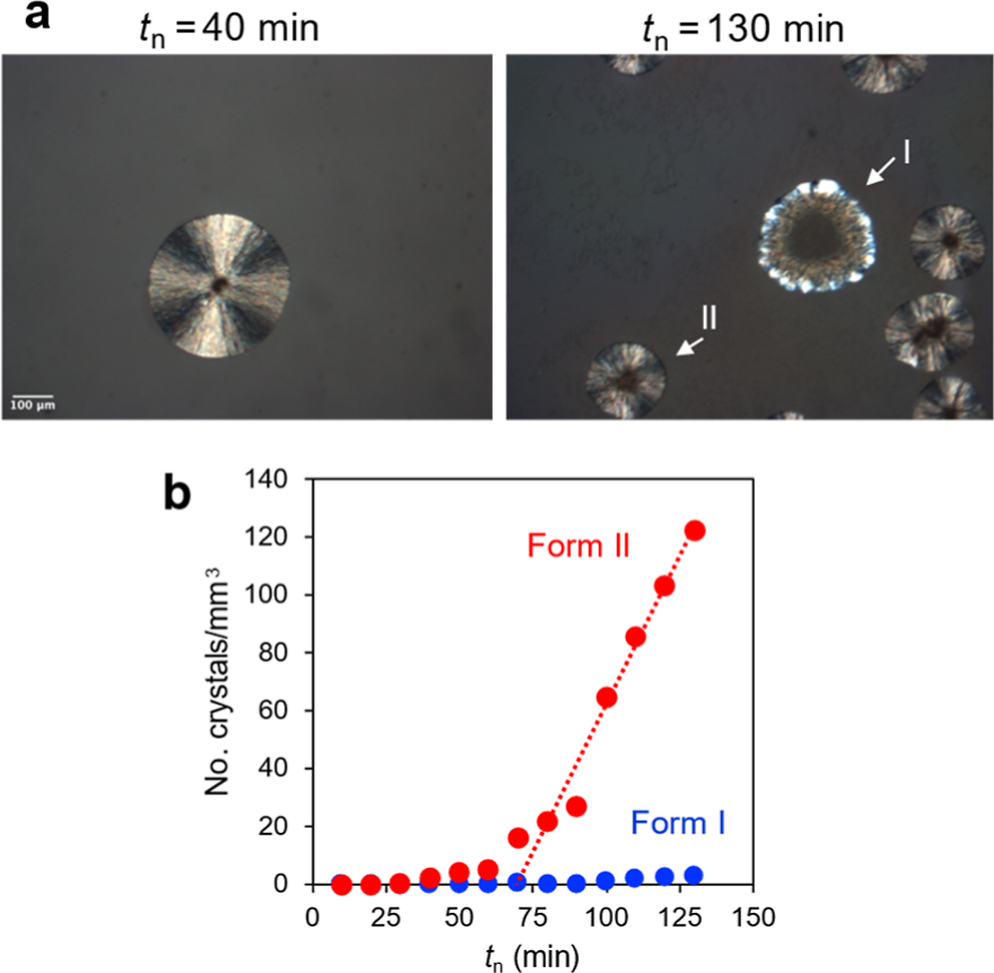
Measuring nucleation rates in POS doped with 10 wt % Span 80 using a two-stage method. (a) Typical images showing higher crystal density after longer nucleation time *t*_n_ at 353 K followed by the same growth time (1 min) at 403 K. (b) Crystal density plotted against *t*_n_. The line indicates the slope at the steady state, which is the nucleation rate.

Figure 12 shows the rates of crystal nucleation and growth at 353 K in a POS melt doped with Span 80. Figure 12a shows that with increasing Span 80 concentration, the Form I nucleation rate decreases, while the Form II nucleation rate increases and appears to plateau. Figure 12b shows that both polymorphs grow faster as Span 80 concentration increases. As in the case of POS doped with Tween 80 (Figure 7), POS doped with Span 80 does not show the simplicity of proportional changes of the nucleation rates and the growth rates, which we discuss later.

**Figure 12:**
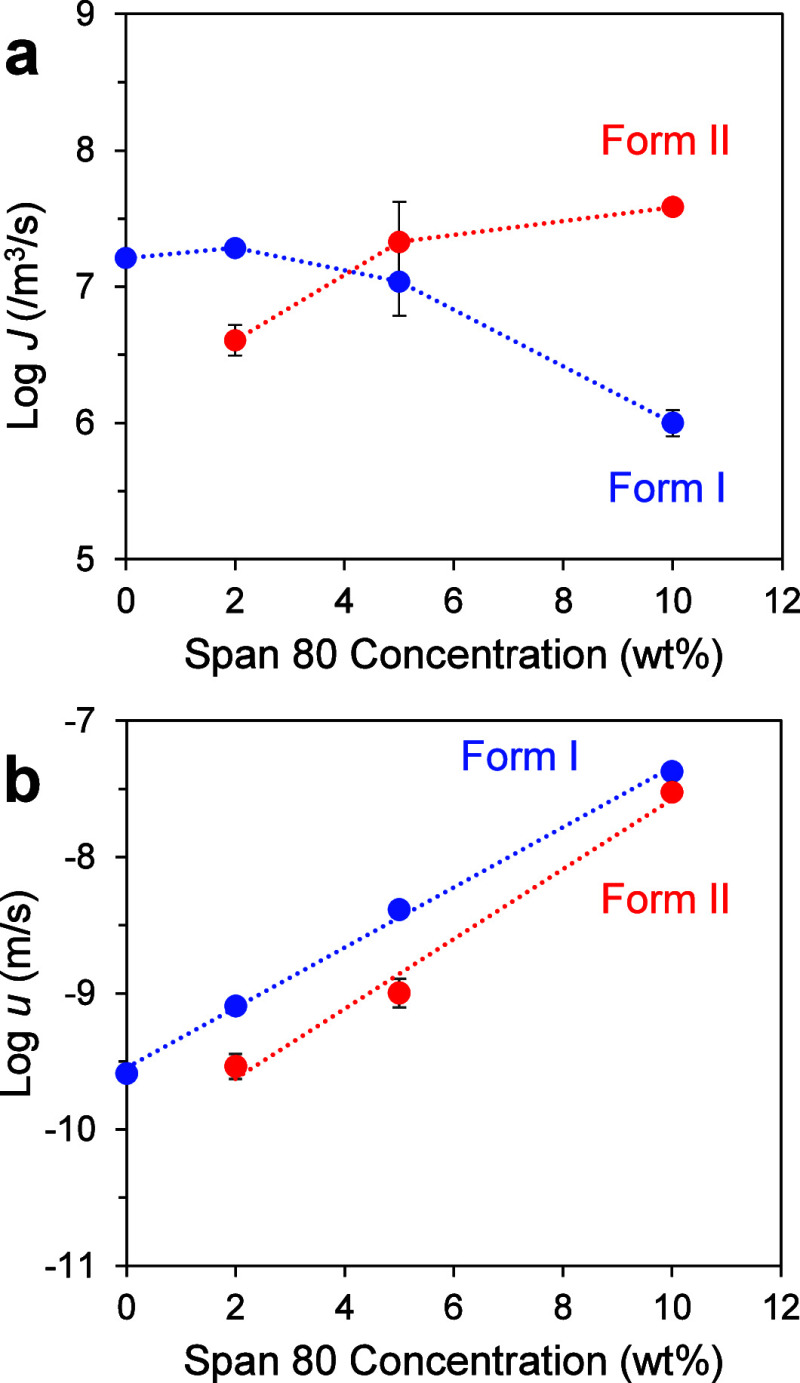
(a) Nucleation rates and (b) growth rates of POS polymorphs at 353 K as functions of Span 80 concentration.

Figure 13 presents the “thickness test” to determine whether the nucleation process observed is a volume process or surface mediated. This test was conducted in the same way as described above for Tween 80 as the dopant, apart from a different nucleation time (120 min in this case). Figure 13 shows that the number of crystals observed is proportional to the liquid-film thickness, and the trend holds for both Form I and Form II. These results indicate that the nucleation process observed is a volume process and justifies its analysis later as a homogeneous nucleation process.

**Figure 13:**
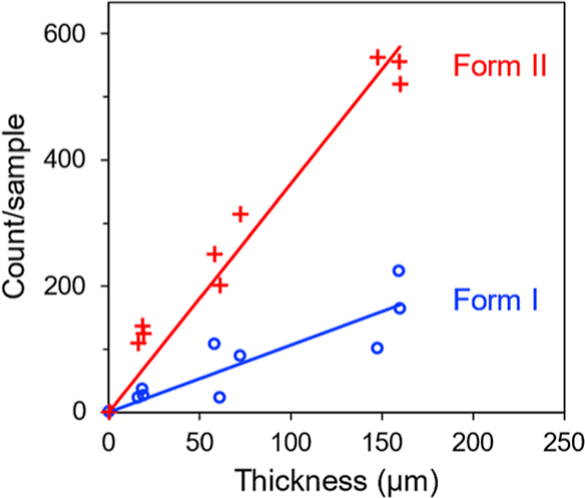
Thickness test to demonstrate that crystal nucleation observed in POS doped with 10 wt % Span 80 is a volume process.

To relate the observed nucleation rates with the polymorphic outcome of crystallization, a POS melt doped with 10 wt % Tween 80 or Span 80 was nucleated at 353 K and heated to 403 K to fully crystallize it, and the resulting crystals were analyzed by PXRD for polymorphic identification. The time of nucleation was 100 min for POS containing 10 wt % Tween 80 and 130 min for POS containing 10 wt % Span 80. According to the measured nucleation rates, the Tween 80 sample should yield a mixture of Forms I and II, and the Span 80 sample predominantly Form II. Figure 14 shows the PXRD patterns of the products of this experiment, along with the reference patterns for the two polymorphs (CSD Refcode YIMVUO for Form I^40^ and YIMVUO01 for Form II^31^). The Tween 80 sample yielded a mixture of Forms I and II and the Span 80 sample only Form II within the limit of detection, exactly as predicted. For pure POS, Form I was observed, confirming the previous report.^31^ These results demonstrate the internal consistency of our findings and the possibility to utilize the nucleation and growth rates to predict the polymorphic outcome of a crystallization process.

**Figure 14:**
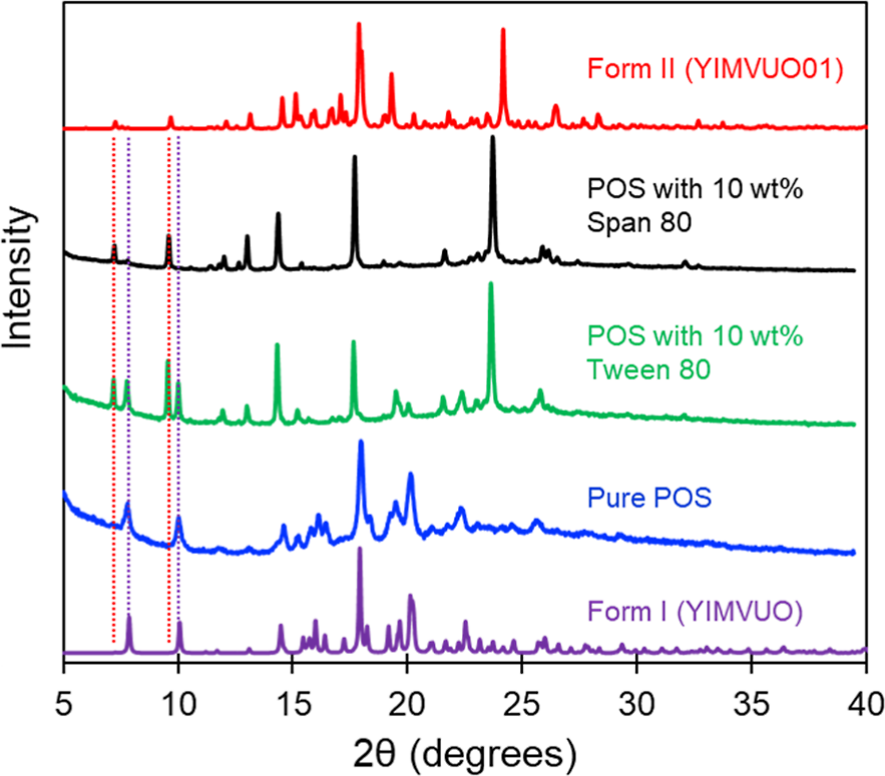
PXRD traces of (top to bottom) Form II simulated pattern (CCDC reference YIMVUO01), POS + 10 wt % Span 80, POS + 10 wt % Tween 80, Pure POS, and Form I simulated pattern (CCDC reference YIMVUO). Dotted lines indicate unique peaks used for polymorph identification.

## Discussion

This study has investigated the crystallization and polymorphic selection in a POS melt doped with two structurally related surfactants, Tween 80 and Span 80. The two surfactants similarly enhance the crystal growth rates of both polymorphs of POS, but have different effects on their nucleation rates, without exhibiting the proportional-change behavior observed for other binary systems.^15,21–23^ In lacking the proportional-change behavior, the surfactant-doped POS stands in contrast to the polymer-doped POS, which exhibits this behavior.^23^ Furthermore, while the pure melt preferentially nucleates Form I,^31^ the doped melts have greater propensity to nucleate Form II. Here we discuss these results.

We first show that with the simple assumption of ideal mixing, the CNT gives a satisfactory account for the nucleation kinetics in the surfactant-doped POS melts, without invoking specific guest–host interactions, and that the lack of the proportional-change behavior stems from the effects of the surfactants on the thermodynamic factor in the CNT. Previous work has tested the CNT by applying it to crystal nucleation in a pure melt,^19,20,23^ and here we provide a stronger test by simultaneously applying it to pure and doped melts. The essence of this test is to vary the driving force for crystallization, Δ*G*_v_, by changing the temperature of the pure melt (Figure 1) and by changing the concentration of the doped melt, and to observe whether the nucleation rate changes according to the CNT.

For a binary melt, Δ*G*_v_ is calculated from

2 $$\Delta G_{v}=RT\;\ln\left(x/x_{0}\right)$$

where *x* is the mole fraction of POS in a supersaturated solution and *x*_0_ is its solubility in mole fraction. *x*_0_ is given by

3 $$RT\;\ln x_{0}=-\left(G_{L}-G_{C}\right)$$

where (*G*_L_ – *G*_C_) > 0 is the driving force of crystallization in a pure POS melt, whose calculation has been described above in connection with Figure 1d. Equations 2 and 3 are valid for ideal solutions.

Figure 15 shows the log­(*J*/*u*) vs 1/(*T*Δ*G*_v_^2^) plot for the surfactant-doped melts, in analogy to the plot in Figure 1d for the pure POS melt. The plots are approximately linear, indicating that the CNT accurately describes our data. Remarkably, for each polymorph (I or II), a single trend is observed for the nucleation kinetics measured in the presence of Tween 80 and Span 80. This indicates that in terms of their effects on the nucleation kinetics, both surfactants act as “ideal diluents”, interacting with the host molecules as in an ideal solution. From the slopes of the CNT plots in Figure 15, we obtain σ_I_ = 0.019 J/m^2^ and σ_II_ = 0.012 J/m^2^ for Form I and Form II nucleating from the doped melts, respectively. Again, each σ value applies to the melt doped with both surfactants as it came from fitting the combined data. Thus, despite their different structures and properties (Table 1), the two surfactants have a similar effect on the nucleus/liquid interfacial tension.

**Figure 15:**
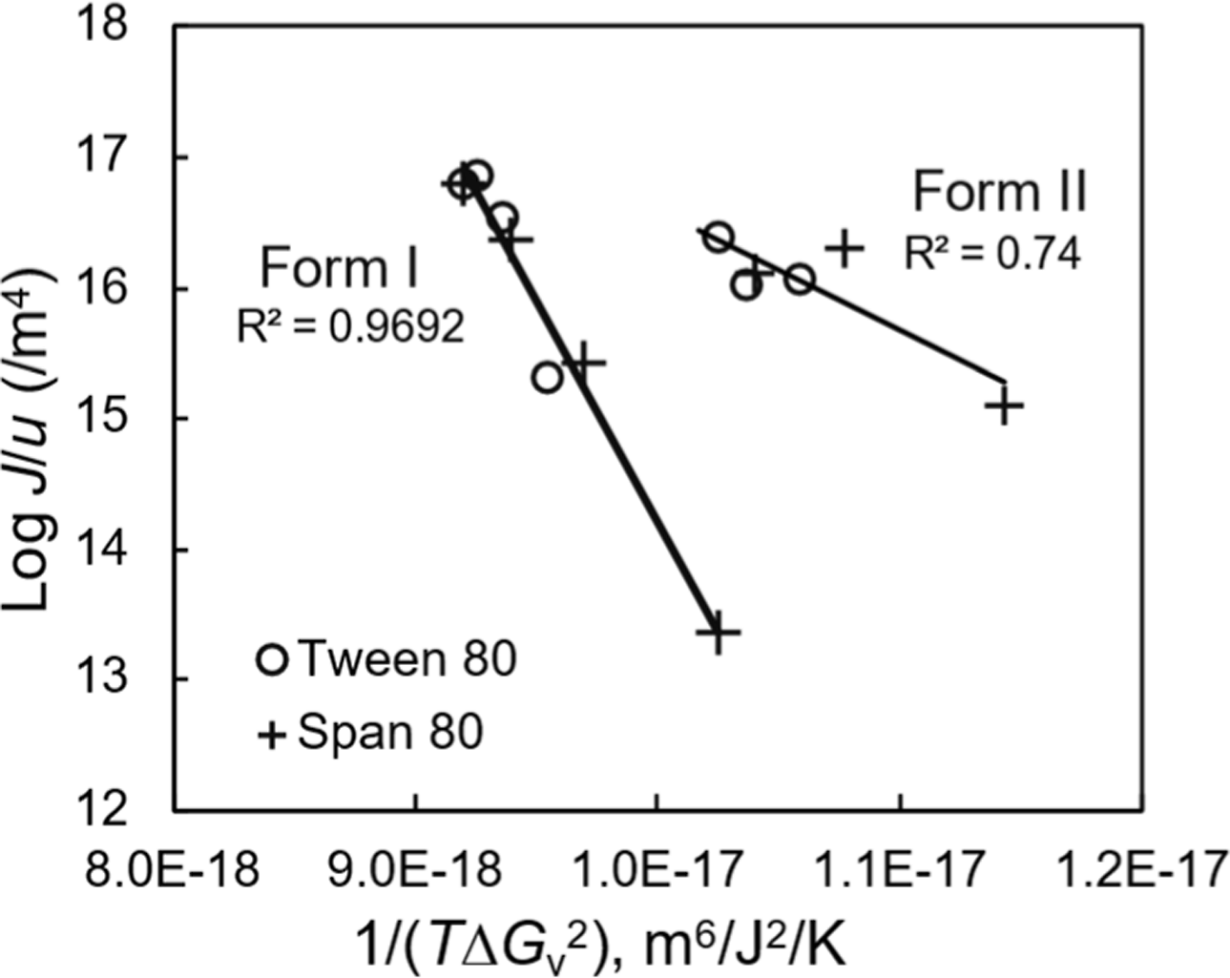
Test of the CNT for describing crystal nucleation in a POS melt containing Tween 80 or Span 80.

For the nucleation of Form I, the presence of a dopant increases the σ value relative to the pure melt: σ_I_ = 0.012 J/m^2^ for nucleation from the pure melt (Figure 1d); σ_I_ = 0.019 J/m^2^ for nucleation from the doped melts (Figure 15). This could be attributed to the greater structural difference between the crystal and the liquid, since the liquid now contains an impurity, but the crystal nucleus is presumably chemically pure. We cannot make this comparison for Form II since its nucleation rate in the pure melt is presently unknown. In contrast to the trend observed here, σ (pure) < σ (doped), the opposite trend was reported by Zhang et al. for polymer-doped fluconazole based on variable-temperature data and a different format of the CNT analysis.^22^

It is noteworthy that in the presence of Tween 80 or Span 80, σ_I_ is larger than σ_II_ (0.019 vs 0.012 J/m^2^). To understand this, it would be informative to know the corresponding rank order in the pure melt, but this is presently unknown since the value for Form II is lacking. This is a puzzling result in light of the crystal structures of the two polymorphs (Scheme 1). These structures might suggest σ_I_ < σ_II_ since Form I contains molecular layers in which the molecules are antiparallel, whereas Form II contains molecular layers in which the molecules are parallel, and presumably the liquid contains molecules in random orientations and thus more closely resembles Form I. Furthermore, Form I has lower density than Form II (1.321 vs 1.351 g/mL at 100 K),^31^ suggesting its structure is more similar to the liquid, which should have even lower density. MD simulations could shed light on this puzzle. Together, the rank order σ_I_ > σ_II_ in the doped melts and the increase of σ_I_ by the surfactants, σ_I_ (pure) < σ_I_ (doped), is consistent with the switch of the fast-nucleating polymorph from Form I in the pure melt to Forms II or a mixture of I and II in the doped melts.

## Concluding Remarks

This study investigated the effects of two structurally related surfactants on the crystal nucleation and growth in a POS melt. Between 0 and 10 wt %, the two surfactants similarly enhance the growth rates of POS’s two polymorphs, but have different effects on their nucleation rates, without exhibiting the proportional-change effect observed for several binary systems.^15,21,22^ The CNT provides an accurate description of the observed nucleation rates using the crystal growth rate to represent the kinetic factor and the crystallization driving force calculated on the assumption of ideal mixing. To our knowledge, this is the first stringent test of the CNT by simultaneously applying it to pure and doped melts where the driving force for crystallization is varied by temperature in the pure system and by concentration in the doped system. The breakdown of the simple proportionality between nucleation and growth rates stems from the significant influence of the surfactants on the driving force of crystallization Δ*G*_v_ and in turn the thermodynamic factor in the CNT. Surprisingly, the two surfactants influence the nucleation process in a similar way, allowing the modified nucleation rates to be fitted together. This analysis finds a higher nucleus/liquid interfacial energy for the nucleation of Form I from a doped melt (0.019 J/m^2^) than Form II (0.012 J/m^2^) and shows that the dopants increase the interfacial energy between a Form I nucleus and the surrounding liquid, perhaps by increasing the structural difference between the chemically pure crystal and an impure melt. Together, these effects account for the change of polymorphic preference from Form I in the pure melt to Forms I and II in the doped melts.

A surprising result from this work is that the two surfactants have significantly different HLB values and assembly behaviors, but similar effects on the nucleation process in a POS melt. In the CNT framework, they behave as ideal diluents and the doped melts have the same nucleus/liquid interfacial energy for a given polymorph, independent of the surfactant. This argues that the common picture that a surfactant preferentially populates an interface does not describe its interaction with a nucleus/liquid interface. Had the surfactants been enriched at the nucleus/liquid interface, there should be a significant difference between Tween 80 and Span 80 in their effect on nucleation kinetics. The simplicity observed here is analogous to “water-activity controlled ice nucleation” where the differences between various solutes vanish when their effects on ice nucleation temperature are compared at the same water activity.^41^ When compared at the same Δ*G*_v_ (i.e., the same POS activity), Tween 80 and Span 80 have the same influence on the nucleation rate of the host molecules. This simplicity, if general, could help predict the effect of a second component on the crystal nucleation rate.
